# Dumpster Diving in the Emergency Department: Quantity and Characteristics of Waste at a Level I Trauma Center

**DOI:** 10.5811/westjem.2020.6.47900

**Published:** 2020-08-24

**Authors:** Sarah Hsu, Cassandra L. Thiel, Michael J. Mello, Jonathan E. Slutzman

**Affiliations:** * Warren Alpert Medical School of Brown University, Providence, Rhode Island; † New York University Grossman School of Medicine, Department of Population Health, New York, New York; ‡ Warren Alpert Medical School of Brown University, Department of Emergency Medicine, Providence, Rhode Island; § Massachusetts General Hospital and Harvard Medical School, Department of Emergency Medicine, Boston, Massachusetts

## Abstract

**Introduction:**

Healthcare contributes 10% of greenhouse gases in the United States and generates two milion tons of waste each year. Reducing healthcare waste can reduce the environmental impact of healthcare and lower hospitals’ waste disposal costs. However, no literature to date has examined US emergency department (ED) waste management. The purpose of this study was to quantify and describe the amount of waste generated by an ED, identify deviations from waste policy, and explore areas for waste reduction.

**Methods:**

We conducted a 24-hour (weekday) ED waste audit in an urban, tertiary-care academic medical center. All waste generated in the ED during the study period was collected, manually sorted into separate categories based on its predominant material, and weighed. We tracked deviations from hospital waste policy using the hospital’s Infection Control Manual, state regulations, and Health Insurance Portability and Accountability Act standards. Lastly, we calculated direct pollutant emissions from ED waste disposal activities using the M+WasteCare Calculator.

**Results:**

The ED generated 671.8 kilograms (kg) total waste during a 24-hour collection period. On a per-patient basis, the ED generated 1.99 kg of total waste per encounter. The majority was plastic (64.6%), with paper-derived products (18.4%) the next largest category. Only 14.9% of waste disposed of in red bags met the criteria for regulated medical waste. We identified several deviations from waste policy, including loose sharps not placed in sharps containers, as well as re-processable items and protected health information thrown in medical and solid waste. We also identified over 200 unused items. Pollutant emissions resulting per day from ED waste disposal include 3110 kg carbon dioxide equivalent and 576 grams of other criteria pollutants, heavy metals, and toxins.

**Conclusion:**

The ED generates significant amounts of waste. Current ED waste disposal practices reveal several opportunities to reduce total waste generated, increase adherence to waste policy, and reduce environmental impact. While our results will likely be similar to other urban tertiary EDs that serve as Level I trauma centers, future studies are needed to compare results across EDs with different patient volumes or waste generation rates.

## INTRODUCTION

Healthcare facilities create significant amounts of waste, with a majority of medical supplies disposed after a single use. Healthcare facilities in the United States (U.S.) generate over 6600 metric tons of waste each day, which is approximately 19 kg per patient per day and 2 million tons of waste each year.^1^,^2^ This makes healthcare the second largest industry to contribute to landfill waste (only the food industry generates more).^3^ Overall, the US healthcare industry contributes nearly 10% of all US greenhouse gas emissions as well as additional other pollutants that adversely affect human health.^4^ Healthcare waste is a direct contributor to these emissions and an important indicator of the impact of procurement practices.

Improper sorting of medical wastes can increase healthcare costs. Overuse of hazardous waste disposal increases the costs and footprint of hauling and treating normal solid wastes. However, underuse of hazardous waste disposal can pose a public health risk and may incur fines. In some clinical spaces, even unused items must be thrown away after a patient has been treated in the space, adding not only to the footprints and costs of disposal, but also to the purchase of unnecessary, wasted supplies. Prior studies have shown that there is poor segregation of healthcare waste into proper waste streams, and there are many opportunities to divert waste from landfills, such as recycling and single-use device reprocessing for reuse.^5^–^7^ Efforts to reduce healthcare waste generation and improve sorting practices have the potential to reduce the environmental impact of healthcare and significantly reduce hospital costs for waste hauling and supply procurement.^8^

A waste audit is an effective way to visualize the categories of waste generated in a healthcare facility, locate where and how different types of waste are processed, and identify areas for waste diversion or waste management improvement. While waste audits of entire hospitals, intensive care units, operating rooms, and specific surgical procedures have been conducted previously, there is no literature surrounding waste management of the emergency department (ED) in the US.^6^–^10^ As a result, little is known about the quantity and characteristics of ED waste. However, the ED represents a significant portion of US healthcare, generating approximately 140 million visits annually, nearly 5% of all healthcare expenditures, and 12% of all outpatient visits.^11^,^12^ Understanding the characteristics of ED waste allows hospitals to find opportunities to reduce waste disposal costs and reduce the environmental impact of emergency care.

The objective of this study was to quantify and describe the amount of waste generated by an ED, identify deviations from local waste management policies and guidelines, calculate direct pollutant emissions from waste disposal practices, and explore areas for waste diversion and reduction.

## METHODS

### Study Design and Setting

We conducted a 24-hour physical waste audit in the ED of an urban, tertiary-care academic medical center. The medical center is a Level I trauma center with an annual ED volume of approximately 110,000 patient encounters per year. Following standard practice, Environmental Services staff collected all municipal solid waste (MSW), regulated medical waste (RMW), and recycling waste between 11 pm July 25 and 11 pm July 26, 2019. RMW included all items thrown in red biohazard bags as well as filled sharps containers. Recycling waste included all items in recycling bins and all paper with protected health information (PHI) disposed of in secure bins.

**Population Health Research Capsule**

What do we already know about this issue?

Healthcare contributes 10% of United States’ greenhouse gases and two million tons of waste each year. No literature on emergency department (ED) waste management currently exists.

What was the research question?

*What are the quantities, characteristics, and carbon footprint (CO_2_e) of ED waste?*

What was the major finding of the study?

*The ED produced 672 kilograms (kg) waste/day and 1.99 kg waste/patient. Waste disposal itself emitted 3 metric tons CO_2_-e/day.*

How does this improve population health?

Pollution and climate change harm human health. Reducing ED waste may reduce upstream and downstream pollution, mitigate climate change effects, and save money.

All waste described during the study period was stored in a designated collection space. The waste was then sorted into separate categories based on predominant material and subsequently weighed. The waste categories we selected were the following: hard plastic; soft plastic; paper products; food waste; textiles; glass; metal; electronic waste; and unused items/mixed materials. All unused items (defined as unopened items or opened but unused items) and uneaten food were indexed and counted. Any loose sharps found were also segregated and weighed as mixed material, but due to safety reasons were not counted. All pulse-oximetry probes found in the waste were also counted due to the institutions’ ability to send them for single-use device reprocessing, a process regulated by the US Food and Drug Administration to allow for single-use devices to be cleaned, repaired (if needed), re-sterilized (when indicated), inspected, and re-packaged for clinical use.^13^ Pharmaceutical wastes are handled by the hospital’s pharmacy department and were excluded. No universal wastes were collected during the study period.

We identified deviations from hospital waste policy by using definitions from the hospital’s Infection Control Manual and Health Insurance Portability and Accountability Act standards. In addition, since RMW has been regulated on a state level since the US Medical Waste Tracking Act of 1988 expired in 1991, we defined RMW using state regulations. State regulations define medical waste as blood and blood products (including draining, liquid state, and materials saturated or dripping with blood); pathological waste (including human anatomical parts and specimens of body fluids, excluding urine, nasal secretions, sweat, sputum, vomit, or fecal matter that don’t contain visible blood or confirmed diagnosis of infectious disease); cultures and stocks of infectious agents and associated biologicals; contaminated animal waste; sharps (including medical items that can cause punctures or cuts); and biotechnology by-product effluents.^14^ Such waste must be rendered safe (for example, via autoclave and shredding sharps) and then may be disposed with MSW. Otherwise, it must be handled by certified haulers to be treated off-site.

The primary author (SH) was present and supervised all waste sorting and weighing, which was completed with the assistance of the senior author (JES) and three research assistants. Any disagreements regarding appropriate waste category were resolved by consensus. All study personnel wore strict isolation personal protective equipment throughout the waste audit, and sharps containers were weighed “as-is” without opening and sorting any of their contents. Upon completion of the waste sorting, all waste was disposed in compliance with hospital policies.

This project was undertaken as a quality improvement initiative at our subject hospital, and as such was not formally supervised by the institutional review board per its policies.

### Measurements

All categories and types of waste were weighed using an Edlund ERS-60 Digital Receiving Scale with a sensitivity of 0.005 kilograms (kg). ED administrative staff provided aggregate data on patient volume and total length of stay during the 24-hour period of study and for total fiscal year 2019 for normalization purposes.

### Data Analysis

Data were entered into and analyzed using Excel (Microsoft, Redmond, WA), with univariate analysis listing frequency counts and percentages. To obtain estimates of annual waste generation rates, we normalized data collected by number of patient encounters, number of patient-hours in the ED, and by time, and we subsequently extrapolated by totals of those values for the fiscal year.

We estimated direct pollutant emissions from waste disposal activities using the M+WasteCare Calculator (Mazzetti, San Francisco, CA.), specifying that MSW was landfilled, RMW was autoclaved and then landfilled, and recyclables were sent for recycling. M+ Wastecare calculates the approximate pollutant load associated with each step in the waste’s journey for each pollutant.^15^ These pollutant loads are added to give a final amount, in carbon dioxide equivalent (CO_2_e), the standard unit for carbon footprints. In all cases, emissions factors are used to perform the calculation. This includes (as applicable), emissions associated with transportation to the disposal facility, emissions associated with energy used for disposal (for autoclaving and alternative sources), emissions associated with transportation of any residuals to landfill, and emissions associated with landfill.

## RESULTS

Over the 24-hour period of our study, we collected a total of 671.785 kg of waste (Table 1), or 1.999 kg/patient encounter. Of this total, 84% (567.38 kg) was collected in MSW (clear) bags, 11% (71.665 kg) in RMW (red biohazard) bags, and 5% (32.74 kg) in recycling bins. Excluding sharps containers, which were not individually audited for safety reasons, only 15% (7.45 kg) of the waste disposed in red bags met the criteria for RMW. Assuming all contents of sharps containers were correctly disposed, less than 5% (31.015 kg) of total waste was true RMW. Similarly, less than 5% (32.74 kg) of all waste was disposed in recycling bins. The majority, 86% (28.011 kg), of recycling waste consisted of paper records with PHI thrown in secure bins. Excluding the paper containing PHI, 20% (0.95 kg) of waste thrown into the recycling bins was not recyclable.

The predominant material found in both MSW and RMW was plastic, at 65% of total waste, 71% (400 kg) of MSW, and 43% (31 kg) of RMW. The second most abundant waste category in MSW and RMW was paper products: 16% (92.43 kg) of MSW and 4% (3.105 kg) of RMW. Of all paper product waste, only 23% (28.011 kg) was shredded and recycled through the PHI paper secure bin. The third largest category in MSW and RMW was food waste, totaling over 41 kg, or 6% of the total. Within food waste, 19% (over 8 kg) was unopened or uneaten food, such as diet cranberry juice, bananas, and milk cartons, most of which are food items found in brown-bag meals given to patients.

Several large-quantity items in MSW and RMW were also sorted and weighed. There were 6.35 kg of emesis basins; 2.96 kg of tourniquets, of which 420 grams (g) or 76 tourniquets were still bundled and unused; 43.63 kg of gloves; and 24.395 kg of disposable cups. We found 201 unused items (5.92 kg), such as normal saline syringes, intravenous (IV) catheters, electrocardiogram and monitor electrode packets, and IV fluid bags in both MSW and RMW bags. Additional data regarding the breakdown of solid, medical, and recycling waste is in the supplementary appendix.

Base case pollutant emissions resulting per day from waste disposal include 3110 kg CO_2_e (71% from RMW, 29% from MSW, and <1% from recycling) and 576 g of other criteria pollutants, heavy metals, and toxins (84% RMW, 13% MSW, and 3% recycling). These greenhouse gas emissions are equivalent to driving a car 7700 miles and only represent the pollution from the disposal of waste, not including the upstream environmental costs of their production, distribution, and use.^16^

We identified several deviations from institutional waste policy. We found paper products with PHI in MSW and RMW, which should have been placed in the PHI-paper secure bin. There were 285 g of loose sharps in standard red bags rather than being placed in sharps containers, which would have accounted for 1.9% of total sharps waste (assuming all contents of sharps containers were actual sharps). In addition, 29 pulse-oximeter probes that should have been diverted and sent for re-processing were found in both MSW and RMW.

Extrapolating our one-day data to a full year, our subject ED is estimated to generate 194,163 – 245,202 kg of waste annually (see Table 2).

## DISCUSSION

In this 24-hour waste audit of an academic, tertiary-care ED, we collected, sorted, characterized, and weighed 672 kg of waste, representing 1.999 kg/patient. To the best of our knowledge, this is the first documented waste audit of an ED in the US and represents an important start in describing and improving upstream and downstream environmental impacts of the emergency care we provide.

Little is known about the quantity and characteristics of ED waste in the US, and the only prior studies of ED waste have been conducted in Jordan and Australia. Two audits of EDs in Jordan published in 2004 and 2007 revealed that the daily generation rate per patient ranges from 0.289 – 0.479 kg/patient/day, lower than the findings of our study.^17^,^18^ Comparing to our institution, though, is challenging given the likely large differences in operations in a non-Organization for Economic Cooperation and Development country. In 2019, a study detailing a pilot program to reduce ED waste in a regional Australian hospital did not audit the waste, but found that efforts to improve waste segregation and recycling failed due to poor compliance.^19^ Staff felt that the process was time consuming and complicated and environmental services staff were seen mixing different waste bins together to simplify the process.

In our study, 85% of all waste thrown into RMW did not meet the criteria for RMW. Given that RMW costs 5–10 times as much to dispose of compared to solid waste, diverting non-RMW from the red biohazard bags is a significant opportunity for cost savings. While our waste audit revealed that over 10% of total ED waste was disposed of as RMW, the Centers for Disease Control and Prevention (CDC) suggests that only 3–5% of hospital waste requires disposal as RMW.^20^ However, if all non-medical waste were diverted from the RMW bags, the percentage of true RMW, including sharps, would be 4.6%, within the range of the CDC criteria.

The Healthcare Plastics Recycling Council estimates that approximately 20–25% of healthcare waste is plastic.^21^ Another study published in 2003 that looked at waste in a Massachusetts hospital revealed that only 20% of solid waste was plastic.^22^ However, a total of 65% of ED waste in our study was plastic, higher than both estimates. This is likely due to the fact that plastics production has been increasing exponentially over the past few decades.^23^ Other studies have shown that plastic composition is highest in the ED and that locations where there is high turnover of patients and poor set-up of bin locations reduces proper waste disposal as a priority.^17^,^24^ This discrepancy is also likely heightened due to the prevalence of single-use disposable devices in the ED. Efforts to explore reusable alternatives can also lead to waste reduction and supply savings.^25^

There is potential for increased recycling in the ED. Assuming all waste made of metal, glass, paper, and hard plastic (that do not meet RMW criteria) are able to be diverted and recycled, up to 258 kg or 38% of all ED waste could be recycled and diverted from landfill waste. Given that nearly 20% of items thrown into existing recycling bins (excluding secure paper bins) was non-recyclable, any efforts to increase recycling in the ED would need to be accompanied by training and other system changes to improve accessibility to recycling bins.

Loose sharps not contained in sharps containers and paper containing PHI were found in both MSW and RMW. In an ED with hundreds of healthcare workers, hundreds of environmental services staff, and thousands of patients, it shouldn’t be surprising to find occasional waste handling deficiencies. Unfortunately, such events can pose a significant health hazard to staff and privacy risk to patients. These findings could expose a healthcare institution to regulatory agency action.

Waste audits of entire hospitals in Turkey, Iran, and Brazil revealed that 17.1–31% of total hospital waste constitutes food or organic waste.^26^–^28^ This fraction of food waste is higher than in our study of the ED likely due to the fact that audits of entire hospitals include cafeteria or kitchen waste. However, the amount of food waste is still significant. Given that the average person eats 905 kg of food a year, one year of food waste from this ED could feed roughly 17 people for a year.^29^

In addition to the upstream pollution embedded in our supply chains, waste disposal itself directly generates pollutant emissions. Looking specifically at greenhouse gas emissions, which lead to climate change, and extrapolating from a one-day sample, our ED’s waste contributes over 1000 tons CO_2_e per year. This is equivalent to the greenhouse gas emissions of driving 200 passenger cars for one year in the US.^16^ There are additional emissions of toxins, criteria air pollutants, and heavy metals totaling over 200 kg annually. These pollutants, including arsenic, cadmium, dioxins, mercury, nitrogen oxides, sulfur dioxide, particulate matter, and volatile organic compounds, all harm human health. And this does not account for the upstream emissions resulting from the manufacture, transport, and use of materials.

Our results indicate that significant improvements can be made to optimize ED waste management in order to reduce total waste generated, emissions from treatment, and waste hauling and treatment costs. If all the metal, glass, and hard plastic were recycled, if all the pulse-oximeter probes were reprocessed, all batteries went to electronic waste, all food waste was composted or diverted (or better yet, reduced), and all unused items were restocked or donated, approximately 305 kg or 45% of waste could have been diverted. Maximally optimizing the waste stream has the potential to divert over 100 tons of ED waste from the landfill each year.

Optimizing the waste stream is only one part of the solution. One of the benefits of a waste audit is understanding the supply chain of the ED, as simple mass balance would dictate that nearly everything that enters the ED as a supply leaves the department with the patient or as waste. Upstream changes, such as switching disposable items to reusable items and researching opportunities for single-use device reprocessing have the potential to reduce the volume of disposables purchased. Given that the single largest category of waste in the ED is soft plastic, most of which is packaging, efforts to order items that use less packaging or purchase commonly used items separately from kits, has a high potential for waste reduction.

## LIMITATIONS

This study has several limitations to its study design. First, our audit was conducted on a single day and the results may not be representative of the full year. During this 24-hour period, 336 new patients were seen in the ED, which is higher than the daily average for fiscal year 2019 of 310 patients per day. As a result, an annual waste generation rate based simply on multiplying our one-day total by 365 days may be an over-estimate. We therefore generated two separate estimates of annual waste generation rates using kg/patient encounter and kg/patient-hour for comparison (see Table 2). This study was also conducted at a single site. While our results will likely be similar to other urban tertiary EDs that serve as Level I trauma centers, future studies are needed to compare results across EDs in other settings, which may have different patient volumes, waste generation rates, waste sorting practices and policies, and waste hauling and treatment contracts.

For logistical and safety reasons, we limited some of our measurement capabilities. Waste items with liquid contents were classified without regard for the liquid components (i.e., full or incompletely-emptied IV fluid bags were classified as soft plastic). Sharps containers were weighed as-is, with the assumption that all contents were correctly sorted. A visual review of items through the plastic containers confirms this not to be true but could not be quantified due to safety concerns of opening and sorting sharps. Similarly, all true medical waste found in red biohazard bags were not sorted into predominant categories and were calculated as mixed under “unused/mixed” materials in Table 1. All loose sharps in red biohazard bags were weighed in total. PHI-containing paper products incorrectly disposed in MSW were not segregated and weighed out of respect for patient privacy. Pharmaceutical waste was specifically excluded, as it is handled by the pharmacy department and not environmental services at our facility; however, the quantity was not expected to significantly alter the results presented here.

## CONCLUSION

Overall, the ED generated 2 kg of waste per patient encounter, 672 kg of waste per day, and an estimated 194,000 – 245,000 kg of waste per year. We also found poor segregation of MSW and RMW, and several deviations from institutional waste policies. Our study reveals opportunities to reduce total waste generated, decrease hospital waste costs, and reduce the environmental impact of emergency care.

## Supplementary Information



## Figures and Tables

**Table 1:** Composition of all wastes produced in emergency department in 24-hour period.

	Category of waste			Total

	MSW	RMW	Recycling	
Mass (kg)	567.38	71.665	32.74	671.785
% of total	84.46%	10.67%	4.87%	100.00%
Material				
Hard plastic	110.615	17.79	2.525	130.93 (19.5%)
Soft plastic	289.775	13.305	-	303.08 (45.1%)
Paper	92.43	3.105	28.011	123.546 (18.4%)
Food	40.865	0.62	-	41.485 (6.2%)
Textiles	18.695	4.72	-	23.415 (3.5%)
Glass	6.74	0.175	1.02	7.935 (1.2%)
Unused/mixed	5.065	8.02	0.94	14.025 (2.1%)
Metal	2.415	0.04	0.19	2.645 (0.4%)
Electronic waste	0.78	0.04	-	0.82 (0.1%)
Sharps	-	23.85	-	23.85 (3.6%)

*MSW*, municipal solid waste (landfill); *RMW*, regulated medical waste (includes red bag or hazardous solid waste and sharps); *kg*, kilogram.

**Table 2:** Estimated annual rate of ED waste generation for Fiscal Year 2019.

	Measured daily rate	Measured FY2019 stats	Estimated annual rate
Waste (Kg)/day (d)	671.785 kg/d	x 365 d/y	245,202 kg/y
Waste (Kg)/patient	1.999 kg/patient	x 113,297 patients/y	226,522 kg/y
Waste (Kg)/patient hours (h)	0.244 kg/patient-h	x 853,397 patient-h/y	194,163 kg/y

*MSW*, municipal solid waste (landfill); *RMW*, regulated medical waste (includes red bag or hazardous solid waste and sharps); *kg*, kilogram; *y*, year.
