# Supplementary material for: Dumpster Diving in the Emergency Department: Quantity and Characteristics of Waste at a Level I Trauma Center
Source: West J Emerg Med. 2020 Aug 24;21(5):1211–7. doi: 10.5811/westjem.2020.6.47900 (PMC7514403; doi:10.5811/westjem.2020.6.47900)
Supplement: Supplementary file 1 [file wjem-21-1211-s001.docx]

Supplementary Appendix

Table of Contents

[Supplementary Table 1: Solid Waste Breakdown 1](#_Toc48397239)

[Supplementary Table 2: Large Volume Items in Solid Waste 2](#_Toc48397240)

[Supplementary Table 3: Regulated Medical Waste Breakdown 2](#_Toc48397241)

[Supplementary Table 4: Non-RMW thrown in RMW Breakdown 2](#_Toc48397242)

[Supplementary Table 5: Recycling Waste Breakdown 3](#_Toc48397243)

# Supplementary Table 1: Solid Waste Breakdown

| **Hard Plastic** |  | **110.615** |
| --- | --- | --- |
|  | Emesis Basins | 4.600 |
|  | Hard Plastics | 106.015 |
| **Soft Plastic** |  | **289.775** |
|  | Tourniquets | 2.690 |
|  | Gloves | 42.655 |
|  | Soft Plastics | 244.430 |
| **Paper products** |  | **92.430** |
|  | Cups | 24.265 |
|  | Paper Products | 68.165 |
| **Food** |  | **40.865** |
|  | Food Waste | 32.395 |
|  | Unopened Food | 8.470 |
| **Textiles** |  | **18.695** |
|  | Clothing | 16.370 |
|  | Gauze | 2.325 |
| **Glass** |  | **6.740** |
| **Unused items** |  | **5.065** |
| **Metal** |  | **2.415** |
| **Electronic waste** |  | **0.780** |
|  | Batteries | 0.075 |
|  | Pulse Ox Probes | 0.705 |
| **Total** |  | **567.38** |

# Supplementary Table 2: Large Volume Items in Solid Waste

|  | **Cups** | **Gloves** | **Batteries** | **Pulse Ox Probes** | **Plastic Basins** | **Tourniquets** |
| --- | --- | --- | --- | --- | --- | --- |
| Mass (kg) | 24.265 | 42.655 | 0.075 | 0.705 | 4.6 | 2.69 |
| Estimated quantity | 971 | 8531 | 3 | 41 | 204 | 446 |

# Supplementary Table 3: Regulated Medical Waste (RMW) Breakdown

| **Category** | **Kg** |
| --- | --- |
| **True RMW in RMW Bags (excluding sharps)** | 7.165 |
| **Loose Sharps in RMW Bags (Not in Sharps Containers)** | 0.285 |
| **Sharps (In Sharps Containers)** | 23.565 |
| **Non-RMW in RMW Bags (Soft)** | 40.65 |
| **Total RMW** | 71.665 |

Red = true RMW; Gray = Non-RMW Incorrectly Placed in RMW

# Supplementary Table 4: Non-RMW thrown in RMW Breakdown

| **Hard Plastic** |  | **17.790** |
| --- | --- | --- |
|  | Emesis Basins | 1.750 |
|  | Hard Plastics | 16.040 |
| **Soft Plastic** |  | **13.305** |
|  | Tourniquets | 0.270 |
|  | Gloves | 0.975 |
|  | Soft Plastics | 12.060 |
| **Paper products** |  | **3.105** |
|  | Cups | 0.130 |
|  | Paper Products | 2.975 |
| **Food** |  | **0.620** |
|  | Food Waste | 0.620 |
| **Textiles** |  | **4.720** |
| **Glass** |  | **0.175** |
| **Unused items** |  | **0.855** |
| **Metal** |  | **0.040** |
| **Electronic waste** |  | **0.040** |
|  | ECG leads | 0.015 |
|  | Pulse Ox Probes | 0.025 |
| **Total non-RMW waste thrown in RMW** |  | **40.650** |

# Supplementary Table 5: Recycling Waste Breakdown

| **Category of Waste** | **Plastic (Hard)** | **Glass** | **Metal** | **Cardboard** | **Paper (PHI)** | **Non- recyclable** | **Total** |
| --- | --- | --- | --- | --- | --- | --- | --- |
| **Mass (kg)** | 2.525 | 1.02 | 0.19 | 0.06 | 28.005 | 0.94 | 32.74 |
